# Microstructure and Mechanical Properties of Co_32_Cr_28_Ni_32_._94_Al_4_._06_Ti_3_ High-Entropy Alloy

**DOI:** 10.3390/ma15041444

**Published:** 2022-02-15

**Authors:** Jinquan Guo, Chaozhongzheng Tang, Huan Sheng Lai

**Affiliations:** 1 School of Mechanical Engineering and Automation, Fuzhou University, Fuzhou 350108, China; megjq@fzu.edu.cn (J.G.); n190227094@fzu.edu.cn (C.T.); 2 Fujian Key Laboratory of Force Measurement, Fujian Metrology Institute, Fuzhou 350108, China; 3 Sino-French Institute of Nuclear Engineering and Technology, Sun Yat-sen University, Guangzhou 510275, China

**Keywords:** microstructure, high-entropy alloy, mechanical property

## Abstract

High-entropy alloys have good application prospects in nuclear power plants due to their excellent mechanical properties and radiation resistance. In this paper, the microstructure of the Co_32_Cr_28_Ni_32_._94_Al_4_._06_Ti_3_ high-entropy alloy was researched using metallurgical microscopy, X-ray diffraction, and scanning electron microscopy. The mechanical properties were tested using a Vickers microhardness tester and a tensile testing machine, respectively. The results showed that Co_32_Cr_28_Ni_32_._94_Al_4_._06_Ti_3_ had a single-phase, disordered, face-centered, cubic solid-solution structure and was strengthened by solid solution. The alloy lattice parameter and density were estimated as 0.304 nm and 7.89 g/cm^3^, respectively. The test results indicated that the alloy had satisfactory mechanical properties with yield stress and tensile strength of about 530 MPa and 985 MPa, respectively.

## 1. Introduction

High-entropy alloys (HEAs) are a type of alloys which contain five or more principle alloying elements to stabilize solid solution phases by maximizing configurational entropy. HEAs have been extensively studied since they were first reported in 2004 [1]. The novel design idea underlying HEAs has greatly expanded the composition range and research areas of metallic materials. Owing to the high entropy effect, the alloys tend to form simple microstructures, such as body-centered cubic (BCC), face-centered cubic (FCC), and densely packed hexagonal (HCP) [2,3]. It has been found that some HEAs not only have excellent mechanical properties [4,5], but also excellent thermal stability [5], wear resistance [4,5,6,7], and irradiation resistance. Therefore, HEAs are promising materials for application in nuclear power plants.

The phase composition and mechanical properties of HEAs are determined by the element types and their atomic ratios in the alloy [8]. For transition HEAs, AlCoCrFeNiZr_x_ is known to possess a BCC structure when *x* = 0 [9]; with an increase in the Zr content, the mechanical properties are significantly improved, because the phase composition of the alloy changes from an ordered BCC solid-solution phase to an ordered Laves + BCC phase. The phase composition of the AlCoCrFeNiB_x_ alloy changes from a BCC phase to BCC + FCC two-phase solid solution with an increase in the B content; meanwhile, the hardness and fracture strength increase first and then decrease [10]. CoCrFeNiW_x_ is known to possess a single-phase structure for *x* = 0.2 [11]; the phase composition of the alloy changes from a single-phase to a hypoeutectic phase, and the mechanical properties are significantly improved when the W content is increased. The phase composition of Al_x_CoCrFeNiTi changes from α + Al grains to fine equiaxed crystals when the Al content is increased; in this way, the yield stress is increased, but the elongation is decreased [12]. AlFeCrCoNi only exhibits an FCC structure for a low ratio of Al/Ni. However, for a large ratio of Al/Ni, the alloy exhibits a BCC structure. The hardness increases with the increase in the ratio of Al/Ni [13].

The three-component alloy of CoCrNi has a single FCC structure. Its mechanical properties are better than other alloys in the alloy system, especially at liquid nitrogen temperature [14,15,16], but the strength is relatively low at chamber temperature [16]. The alloy used in this study is a new, unreported alloy with Al and Ti elements added. Therefore, in this paper, the microstructure and tensile properties of Co_32_Cr_28_Ni_32_._94_Al_4_._06_Ti_3_ were researched in order to better understand the microstructure and mechanical properties of CoCrNi HEAs. 

## 2. Experimental Materials and Methods

**Figure 1:**
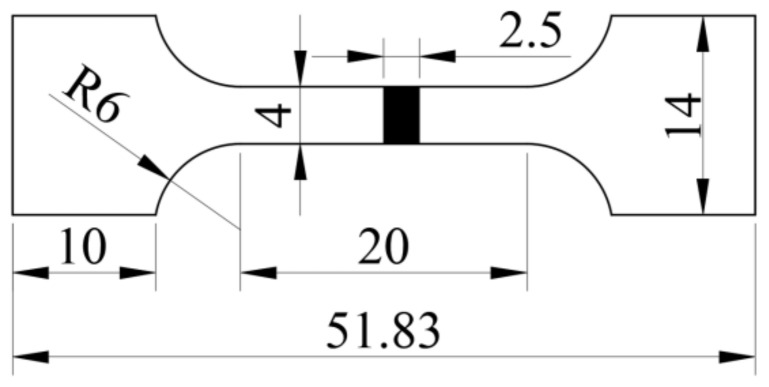
Dimensions of the tensile specimen (mm).

A Co_32_Cr_28_Ni_32_._94_Al_4_._06_Ti_3_ plate with dimensions of 90 mm × 80 mm × 20 mm was prepared using vacuum arc melting equipment with pure metal Co, Cr, Ni, Al, and Ti with purities greater than 99.9 wt%. In order to ensure that the material was not oxidized in the melting process, pure metal Ti was melted firstly to absorb the residual gas of the electric arc furnace. The composition (atomic fraction and weight ratio, %) of the Co_32_Cr_28_Ni_32_._94_Al_4_._06_Ti_3_ used in this study is shown in Table 1. The crystal structure was identified using a DY1602/Empyrean multifunctional X-ray polycrystalline diffractometer (PAnalytical, Alemlo, Holland) with Cu target radiation scanning in the range from 20° to 90° at a rate of 2°/min in a working voltage of 40 kV, a working current of 100 mA, and a characteristic wavelength of 1.5406 Å. The angle measurement accuracy was 0.02°. The HEA density was measured using a drainage method. In the drainage method, the mass of the HEA was measured by a scale; a measuring cylinder was used to measure the volume of water, and the volume of the HEA was the changed volume of the water in the cylinder before and after the HEA was put into the cylinder. The metallography of the HEA was characterized using a MV5000 metallographic microscope (Nanjing Lianchuang Analytical Instrument Manufacturing, Nanjing, China). The Vickers hardness of the HEA was measured using a THV-1MD microhardness tester (Teshi Detection Technology, Shanghai, China) under a load of 200 gf applied for 15 s. The tensile specimens with dimensions of 51.83 mm × 14 mm × 2.5 mm were used, as shown in Figure 1. Three tensile tests were carried out at air room temperature with a strain rate of 1 × 10^−3^ mm/s. The fracture morphology of the tensile specimen was analyzed using a Mira3 ultra-high-resolution field emission scanning electron microscope (FE-SEM) (TESCAN, Shanghai, China). 

## 3. Results and Discussion

**Figure 2:**
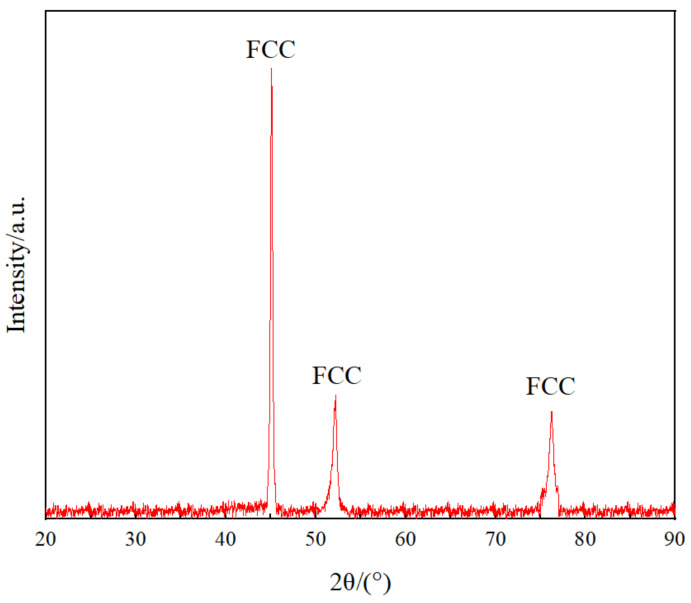
X-ray diffraction (XRD) results of Co_32_Cr_28_Ni_32_._94_Al_4_._06_Ti_3_.

Figure 2 shows the X-ray diffraction (XRD) pattern of Co_32_Cr_28_Ni_32_._94_Al_4_._06_Ti_3_, wherein three diffraction peaks corresponding to the (111), (200), and (220) peaks of the FCC structure were observed. There were no diffraction peaks corresponding to other structures. The result implies that the HEA was only composed of FCC solid solution. 

Generally, Ω > 1.1 and δ < 6.5% are the criteria for determining whether a solid solution phase can be formed [13]. Furthermore, when valence electron concentration (VEC) ≥ 8, the FCC solid solution is considered to be relatively stable [17]. The relevant parameters are calculated as follows:

(1) $$\Delta H_{\mathrm{mix}}=\overset{n}{\underset{i=1,i\neq j}{\sum }}\Delta H_{\mathrm{ij}}^{\mathrm{mix}}c_{i}c_{j}$$

(2) $$\delta =\sqrt{\overset{n}{\underset{i=1}{\sum }}c_{i}{\left({1-r}_{i}/\overset{n}{\underset{i=1}{\sum }}c_{i}r_{i}\right)}^{2}}$$

(3) $$\mathrm{VEC}=\overset{n}{\underset{i=1}{\sum }}c_{i}{\left(\mathrm{VEC}\right)}_{i}$$

(4) $$\Omega =\frac{T_{m}\Delta S_{\mathrm{mix}}}{\left|\Delta H_{\mathrm{mix}}\right|}$$

(5) $$T_{m}=\overset{n}{\underset{i=1}{\sum }}c_{i}{\left(T_{m}\right)}_{i}$$

(6) $$\Delta S_{\mathrm{mix}}=-R\overset{n}{\underset{i=1}{\sum }}\left(c_{i}\ln c_{i}\right)$$

where $\Delta H_{\mathrm{mix}}$ is the total enthalpy of mixing for the system, $\Delta H_{ij}^{\mathrm{mix}}$ is the mixing enthalpy of the atomic pair of the *i*th and *j*th atoms, $c_{i}\;\mathrm{and}\;c_{j}$ are the atomic percentages of elements *i* and *j*, respectively, δ is the difference in atomic radius between the two atoms, $r_{i}$ is the atomic radius of the *i*th element, VEC is the total VEC of the system, ${\left(\mathrm{VEC}\right)}_{i}$ is the VEC of the ith element, Ω is the disorder of the system, $T_{m}$ is the mixed melting point of the system, $\Delta S_{\mathrm{mix}}$ is the mixing entropy of the system, ${\left(T_{m}\right)}_{i}$ is the metal melting point of element *i*, and R is the gas constant.

Table 2 lists the characteristic parameters of the elements of Co_32_Cr_28_Ni_32_._94_Al_4_._06_Ti_3_, and Table 3 lists the mixing enthalpy values of the atom pairs of the HEA elements [18]. According to the calculations, the difference in the atomic size (δ) of the HEA was 4.26%. The disorder of the system (Ω) of the HEA was 3.91, and the VEC of the HEA was 8.1. Therefore, the HEA considered here should be an FCC solid-solution structure in theory. 

The lattice constants of the five elements are listed in Table 4. The lattice constants of alloys can be calculated using the disorder principle [21]:

(7) $$a_{\mathrm{mix}}=\overset{n}{\underset{i=1}{\sum }}c_{i}a_{i}$$

where $c_{i}$ is the atomic percentage of element *i*, and $a_{i}$ is the lattice constants of element *i*. According to the calculations, the lattice constant of Co_32_Cr_28_Ni_32_._94_Al_4_._06_Ti_3_ was 0.304 nm, which was consistent with the lattice constant obtained as per XRD analysis (Table 4). 

The theoretical density of the alloy was calculated using the following formula [22]:

(8) $$\rho_{\mathrm{mix}}=\frac{\sum_{i=1}^{n}c_{i}A_{i}}{\sum_{i=1}^{n}c_{i}A_{i}/\rho_{i}}$$

where $c_{i}$ is the atomic percentage of element *i*, $A_{i}$ is the atomic weight of element *i*, and $\rho_{i}$ is the density of element *i*. From the calculation results, we noted that the theoretical density ($7.85\;g/{\mathrm{cm}}^{3}$) of the HEA was almost identical to the measured value ($7.84\;g/{\mathrm{cm}}^{3}$). It was also found that the HEA results were consistent with the rule of mixtures upon comparing the lattice constant and theoretical density of the alloy, which was a disordered FCC solid-solution structure.

**Figure 3:**
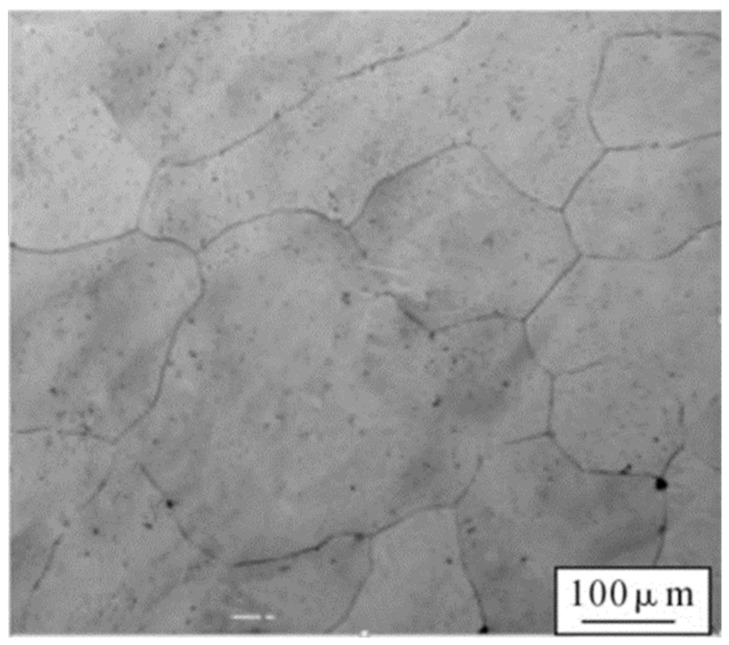
Microstructure of Co_32_Cr_28_Ni_32_._94_Al_4_._06_Ti_3_.

Figure 3 shows a light microscopy image of the metallographic structure of Co_32_Cr_28_Ni_32_._94_Al_4_._06_Ti_3_. As shown in Figure 3, the alloy had a uniform, single-phase, equiaxial crystal structure, and the grain size was about 163 μm as measured using the transection method. 

**Figure 4:**
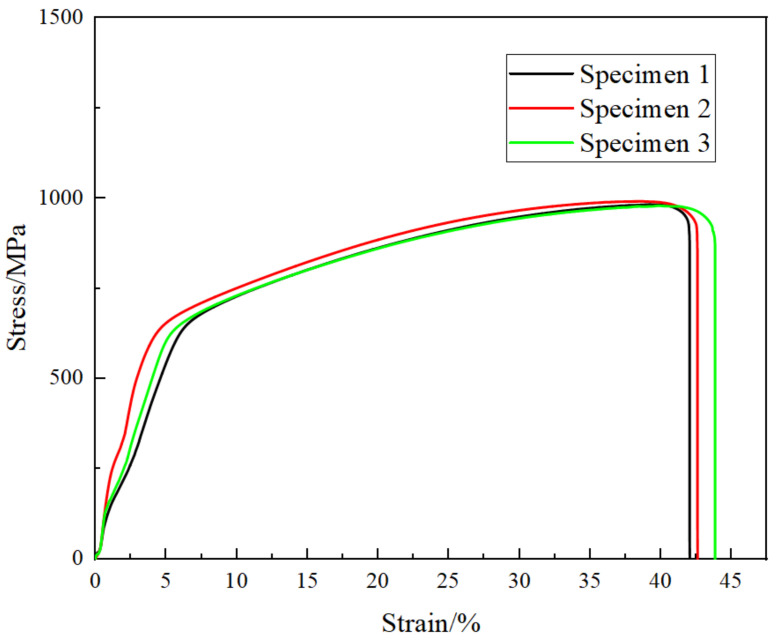
Stress–strain curve of Co_32_Cr_28_Ni_32_._94_Al_4_._06_Ti_3_.

Figure 4 shows the tensile stress–strain curve of Co_32_Cr_28_Ni_32_._94_Al_4_._06_Ti_3_. As shown in Figure 4, the yield strength was 530 ± 6 MPa, the tensile strength was 985 ± 7 MPa, and the elongation was 37.16 ± 0.17%. Compared with the CoCrNi alloy, the yield strength and the tensile strength increased by 103% and 14%, while the elongation decreased by 7%. Obviously, the increase in Al and Ti elements improved the tensile properties of the CoCrNi baselime alloy. Moreover, from Table 5, which compares the uniaxial tensile test results of similar alloys, it can be seen that the alloy affords better strength and plasticity. Moreover, the microhardness of the alloy was 313 HV. Therefore, Co_32_Cr_28_Ni_32_._94_Al_4_._06_Ti_3_ exhibits satisfactory tensile mechanical properties and hardness. 

The tensile properties of the Co_32_Cr_28_Ni_32_._94_Al_4_._06_Ti_3_ studied in this paper are obviously improved compared with N18 and N36 Zircaloy currently used in the nuclear industry, as shown in Table 5. Therefore, the alloy studied in this paper is could be used in the nuclear industry.

Generally, the main strengthening mechanism of a single disordered FCC solid-solution structure is solid-solution strengthening, which mainly originates from the interaction between solute atoms [26].

**Figure 5:**
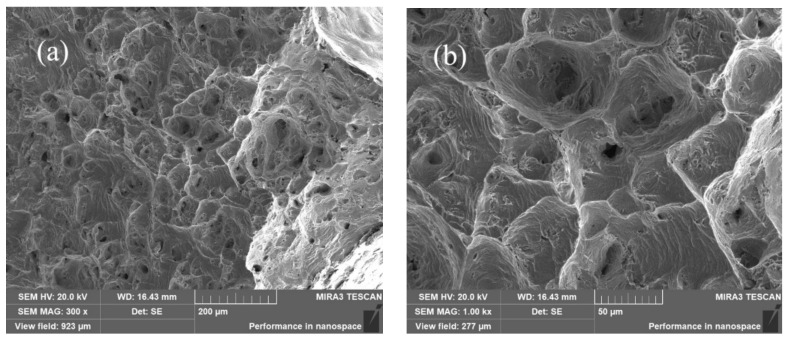
Scanning electron microscopy (SEM) images of the fracture surface of Co_32_Cr_28_Ni_32_._94_Al_4_._06_Ti_3_. (**a**) magnification of 300 times; (**b**) magnification of 1000 times.

Figure 5 shows an SEM micrograph of the fractured tensile specimen of Co_32_Cr_28_Ni_32_._94_Al_4_._06_Ti_3_. A large number of dimples and holes were observed in the fracture. The observed dimples were the traces left on the fracture after micropore nucleation and aggregation, which was the characteristic of micropore aggregation fractures.

The results provide a reference for the study of microstructure and properties of CoCrNi HEAs. In order to fully explain the comprehensive mechanical properties of the HEA, further studies on CoCrNiAlTi HEAs are being carried out, such as the effects of different atomic ratios of major elements on the creep, fatigue properties, and irradiation resistance of CoCrNiAlTi HEAs.

## 4. Conclusions

In this paper, the microstructure and tensile-fracture characteristics of Co_32_Cr_28_Ni_32_._94_Al_4_._06_Ti_3_ were researched. The following conclusions can be drawn:

(1) The Co_32_Cr_28_Ni_32_._94_Al_4_._06_Ti_3_ exhibits a single disordered FCC solid-solution structure with a density of $7.89\;g/{\mathrm{cm}}^{3}$.
(2) The microstructure of Co_32_Cr_28_Ni_32_._94_Al_4_._06_Ti_3_ is equiaxed, with a grain size about 163 μm.
(3) The yield strength, tensile strength, and elongation of the Co_32_Cr_28_Ni_32_._94_Al_4_._06_Ti_3_ are about 530 MPa, 985 Mpa, and 37.2%, respectively. The microhardness of the alloy is 313 HV.

## Figures and Tables

**Table 1:** Atomic and weight ratios of the principal elements of Co_32_Cr_28_Ni_32_._94_Al_4_._06_Ti_3_.

Metal	Co	Cr	Ni	Al	Ti
Atomic ratio (%)	32	28	32.94	4.06	3
Weight ratio (%)	34.11	26.34	34.97	1.98	2.60

**Table 2:** Characteristic parameters of the principal elements of Co_32_Cr_28_Ni_32_._94_Al_4_._06_Ti_3_.

Metal	Co	Cr	Ni	Al	Ti
Melting point (K)	1768.15	2132.15	1728.15	933.15	1941.15
Atomic radius [19,20] (nm)	0.125	0.128	0.123	0.143	0.147
VEC	9	6	10	3	4

**Table 3:** Mixed enthalpies among the principal elements of Co_32_Cr_28_Ni_32_._94_Al_4_._06_Ti_3_ (kJ/mol).

Metal	Co	Cr	Ni	Al	Ti
Co	/	−4	0	−19	−28
Cr	−4	/	−7	−10	−7
Ni	0	−7	/	−22	−35
Al	−19	−10	−22	/	−30
Ti	−28	−7	−35	−30	/

**Table 4:** Densities and lattice constants of the component metals of Co_32_Cr_28_Ni_32_._94_Al_4_._06_Ti_3_.

Metal	Co	Cr	Ni	Al	Ti	Alloy (Calculated)	Alloy (Measured)
Lattice constant (nm)	0.25	0.29	0.35	0.41	0.35	0.30	0.31
Density (g/cm^3^)	8.90	7.19	8.90	2.70	4.54	7.85	7.84

**Table 5:** Comparison of the tensile mechanical properties.

Alloy	Yield Strength (MPa)	Tensile Strength (MPa)	Elongation to Failure (%)
Co_32_Cr_28_Ni_32_._94_Al_4_._06_Ti_3_	530 ± 6	985 ± 7	37.2 ± 0.17
CoCrNi [15]	260	870	40
(Fe_50_Mn_30_Co_10_Cr_10_)_94_C_6_ [23]	450	700	18
CoCrFeNiW_0_._4_ [10]	525	970	11
Al_3_CoCrFeNiTi [11]	115	152	26
CoCrFeMnNi [24]	468	590	30
N18 Zircaloy [25]	390	420	38
N36 Zircaloy [25]	310	520	27

## Data Availability

Not applicable.
